# What controls the observed size-dependency of the growth rates of sub-10 nm atmospheric particles?†

**DOI:** 10.1039/d1ea00103e

**Published:** 2022-03-23

**Authors:** Jenni Kontkanen, Dominik Stolzenburg, Tinja Olenius, Chao Yan, Lubna Dada, Lauri Ahonen, Mario Simon, Katrianne Lehtipalo, Ilona Riipinen

**Affiliations:** Institute for Atmospheric and Earth System Research, University of Helsinki Helsinki Finland jenni.kontkanen@helsinki.fi; Swedish Meteorological and Hydrological Institute Norrköping Sweden; Laboratory of Atmospheric Chemistry, Paul Scherrer Institute Villigen Switzerland; Institute for Atmospheric and Environmental Sciences, Goethe University Frankfurt Frankfurt am Main Germany; Finnish Meteorological Institute Helsinki Finland; Department of Environmental Science (ACES), Bolin Centre for Climate Research, Stockholm University Stockholm Sweden

## Abstract

The formation and growth of atmospheric particles involving sulfuric acid and organic vapors is estimated to have significant climate effects. To accurately represent this process in large-scale models, the correct interpretation of the observations on particle growth, especially below 10 nm, is essential. Here, we disentangle the factors governing the growth of sub-10 nm particles in the presence of sulfuric acid and organic vapors, using molecular-resolution cluster population simulations and chamber experiments. We find that observed particle growth rates are determined by the combined effects of (1) the concentrations and evaporation rates of the condensing vapors, (2) particle population dynamics, and (3) stochastic fluctuations, characteristic to initial nucleation. This leads to a different size-dependency of growth rate in the presence of sulfuric acid and/or organic vapors at different concentrations. Specifically, the activation type behavior, resulting in growth rate increasing with the particle size, is observed only at certain vapor concentrations. In our model simulations, cluster–cluster collisions enhance growth rate at high vapor concentrations and their importance is dictated by the cluster evaporation rates, which demonstrates the need for accurate evaporation rate data. Finally, we show that at sizes below ∼2.5–3.5 nm, stochastic effects can importantly contribute to particle population growth. Overall, our results suggest that interpreting particle growth observations with approaches neglecting population dynamics and stochastics, such as with single particle growth models, can lead to the wrong conclusions on the properties of condensing vapors and particle growth mechanisms.

## Abstract 2

A combination of cluster population simulations and chamber experiments was used to disentangle the factors governing the observed growth rates of atmospheric particles.

## Graphical abstract

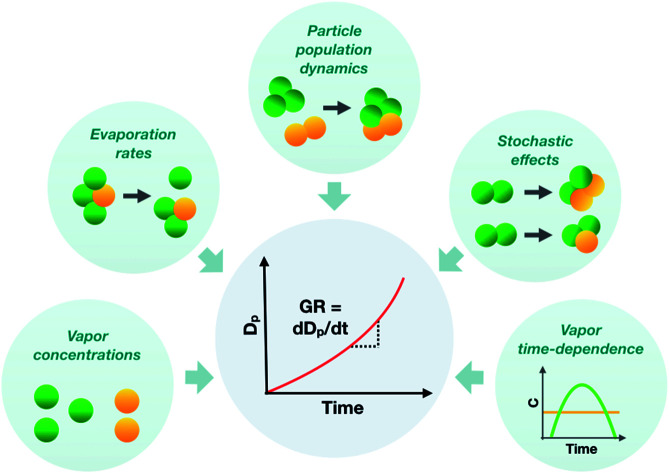

**Environmental significance**

The formation and growth of atmospheric particles involving sulfuric acid and organic vapors can significantly influence the climate, and thus the knowledge of the initial particle growth by these vapors is needed. Particle growth is commonly studied by assessing particle growth rates from measured particle size distributions. Here, we unravel the factors controlling the observed growth rates of sub-10 nm particles, by using cluster population simulations and chamber experiments. We find that particle growth rates are governed by (1) the concentrations and evaporation rates of vapors, (2) particle population dynamics, and (3) stochastic fluctuations. Thus, to get an unbiased view on particle growth mechanisms, observations should be interpreted with approaches able to consider these effects.

## 1. Introduction

The formation and growth of aerosol particles from atmospheric vapors is predicted to produce a significant fraction of the global cloud condensation nuclei, estimates ranging from a few percent up to 80%.^1–5^ Therefore, there is a need for developing robust physical descriptions of this process, which can be implemented in large-scale models. For this, the correct interpretation of the observations on the initial growth of the particle population is necessary.

According to the current understanding, the formation of the first nanometer-sized molecular clusters occurs by nucleation or barrier-less clustering of atmospheric vapors.^6,7^ In the case of nucleation, the molecular clusters on average decay by evaporation faster than grow by collisions of vapor molecules, but stochastic fluctuations enable some of them to reach the critical size regime, where the growth overcomes the evaporation.^7,8^ The stochastics-driven formation can be described by molecular-resolution cluster population models.^9,10^ After the initial formation of clusters, they can continue to grow to larger sizes by condensation of suitable vapors, which can be described by single-particle models that simulate the growth of particle diameter upon condensation.^11,12^ However, the mean size of the particle population is also affected by other population dynamics processes, including the collisions between different molecular clusters or particles and their losses due to coagulation scavenging or other sinks.^13–15^

Due to the limited knowledge of the thermodynamic properties of newly formed molecular clusters and particles, their high evaporation rates are commonly depicted with Kelvin effect, stating that the equilibrium vapor pressure of the compound over the surface of a spherical particle increases with increasing particle size. Therefore, only vapors i with low enough saturation vapor pressure (*p*_sat,i_) (or in the case of mixtures, low enough equilibrium vapor pressure over the mixture) can reach high enough supersaturation to overcome the Kelvin barrier and condense on the smallest particles.^16^

Sulfuric acid is important in the initial steps of particle formation and growth in many environments,^17^ due to its low equilibrium vapor pressure over the mixture of sulfuric acid and bases, such as ammonia and amines.^18^ After the initial growth by sulfuric acid, organic compounds are considered to govern the growth of particles to larger sizes.^19^ Especially, highly-oxygenated organic molecules (HOMs), formed by autoxidation of volatile organic compounds (VOCs), such as monoterpenes, have been observed to participate in particle formation and growth in chamber experiments.^12,20,21^ Organic compounds are commonly divided into different groups based on their volatility.^22^ Extremely low volatility organic compounds (ELVOCs) have very low *p*_sat,i_ and can therefore contribute to particle growth even at the smallest, sub-3 nm, sizes.^12^ Low volatility organic compounds (LVOCs) have higher *p*_sat,i_ and can thus usually drive particle growth only after the particles have grown past a few nanometers.^12,23^

A common approach to study which compounds participate in particle growth based on atmospheric measurements is to (1) deduce the diameter growth rate of particles (in units nm h^−1^) in different size ranges from the time-evolution of particle size distribution^24^ and (2) determine if the observed growth rates can be explained by a condensation mass flux of some vapor (often sulfuric acid) on a single particle,^25^ using measured vapor concentrations. In most environments, particle growth rate is observed to increase with size between ∼1 and 25 nm,^26^ and the condensation of sulfuric acid is found insufficient to explain the growth rates, especially above ∼5 nm.^27–30^ This has been attributed to the important role of oxidized organic vapors, which can condense on particles with increasing efficiency as the particle size increases, due to the diminishing Kelvin barrier.^31^ The process has been proposed to be described by nano-Köhler theory, depicting the activation of particles to growth by organic vapors due to lowering of Kelvin barrier and the solute effect.^32^ However, the nano-Köhler theory can represent the complex dynamics of growing atmospheric particles only under certain ranges of condensable vapor concentrations and the saturation ratio of organic vapors.^33^ Also, comparing the observed particle growth rates with estimates from gas-phase vapors is challenging because of high uncertainties in the measurement of sub-10 nm particle size distribution (which growth rate is calculated from) and in the quantification of condensing vapors.^34,35^

In addition to atmospheric observations, particle growth rates have been studied using chamber experiments, such as the CLOUD (Cosmics Leaving OUtdoor Droplets) experiment at CERN.^12,36,37^ In the CLOUD experiments involving only sulfuric acid and ammonia, growth rate has been found to decrease with size.^36^ This can be explained by the decreasing vapor molecule size relative to particle size as particle size increases, causing the diameter growth rate to decrease with size when evaporation is negligible,^25^ as well as by the reduced influence of van der Waals forces at larger sizes.^36^ In experiments involving only organic vapors, sub-10 nm growth rate has been observed to increase with size.^12,37,38^ The strength of the increase has been shown to depend on the volatility distribution of the organic oxidation products and thus it is influenced for example by nitrogen oxides (NO_*x*_) concentrations,^37,39^ and the specific mixture of organics.^40,41^

In most parts of the atmosphere, sulfuric acid, ammonia, and organic vapors are all present and can be expected to participate in particle formation and growth.^5,17^ Although nucleation involving sulfuric acid, ammonia and HOMs has been investigated in the CLOUD experiment,^42^ the size-dependence of particle growth rate in the presence of all these vapors simultaneously has not been studied in detail.

Besides the properties of the vapors participating in condensation growth, observed sub-10 nm particle growth rates and their size-dependency can also be affected by other factors. These include population dynamics, such as cluster–cluster collisions, coagulation scavenging and the loss of particles onto chamber walls,^13,43^ as well as the time-dependent variation of condensable vapors.^33,44^ In addition, stochastic effects, which are often neglected, can significantly contribute to the growth of the particle population at the smallest sizes.^8,45,46^ The contributions of all these factors influencing observed particle growth rate cannot be separated solely based on measurements, or by using condensation growth models, where the deterministic mass flux of vapor on a single particle is studied. Thus, to interpret observations on sub-10 nm particle growth, modeling methods considering the evolution of the whole cluster or particle population are necessary.^9,14,47^

In this work, we investigate sub-10 nm particle growth by utilizing a combination of experimental data and molecular cluster population simulations. We analyze experiments from the CLOUD chamber involving sulfuric acid, ammonia, and/or oxidation products of monoterpenes,^42^ corresponding to conditions in the boreal forest where particle formation and growth is frequent.^48^ The CLOUD facility enables studying the formation and growth of particles from these vapors under well-controlled, atmospherically relevant conditions with negligible contaminations.^49–51^ To understand the behavior of observed growth rate, we perform molecular-resolution simulations of 1–6 nm cluster and particle population and compare growth rates from experiments to the corresponding simulations. We also apply a metric by Olenius *et al.*^8^ to both experimental and simulation data to assess the impact of stochastic collisions and evaporations on particle growth.

We aim to answer to the following questions: (1) what controls the variation in the observed growth rates of sub-10 nm particles in the presence of one or more condensable vapors, such as sulfuric acid and oxidized organic compounds, and (2) what does this imply in terms of how observed particle growth rates can be interpreted? Especially, we explore the impacts of condensable vapor properties (a) on the size-dependency of observed particle growth rate, (b) on the effects of particle population dynamics, such as cluster–cluster collisions, on particle growth, and (c) on the threshold size above which stochastic effects on particle growth can be neglected.

## 2. Methods

### 2.1 Experiments

#### 2.1.1 Experimental conditions

We use three sets of experiments performed at the CERN CLOUD chamber: (1) pure inorganic experiments with sulfuric acid and ammonia present in the chamber,^36^ (2) pure organic experiments with oxidation products of monoterpenes and NO_*x*_ in the chamber,^37^ and (3) mixed organic–inorganic experiments with sulfuric acid, ammonia, NO_*x*_ and monoterpenes oxidation products in the chamber, resembling the conditions in a boreal forest with some anthropogenic background.^42^ The inorganic experiments were performed in autumn 2017 and the experiments involving organic vapors in autumn 2015. In all the experiments, the temperature of CLOUD chamber was set to 5 °C. For a full list of the experiments and the relevant precursor gas concentrations, see Sect. 3.1.

For the sulfuric acid and ammonia experiments, sulfur dioxide, ammonia and ozone were added to the chamber. Sulfur dioxide and ozone were added up to 5 ppb and 30 ppb, respectively, while ammonia concentration was 41–45 ppt. The photo-oxidation of sulfur dioxide was induced by UV illumination of the chamber resulting in the formation of sulfuric acid and subsequent new particle formation and growth. The sulfuric acid concentration during the experiments was controlled by the UV intensity. For more details, see Stolzenburg *et al.*^36^

For the experiments involving organic vapors, alpha-pinene or a mixture of alpha-pinene and delta-3-carene were added to the chamber together with NO_*x*_. The experiments were typically initiated by switching between neutral and so-called galactic cosmic ray conditions, by switching off the electric field of the chamber and hence allowing ions to be produced in the chamber at an ion-pair production rate of ∼3 cm^−3^ s^−1^. This increases the nucleation rate in the system significantly^42,52^ and results in the formation of a new particle mode at rather constant gas-phase precursor concentrations. The new particle mode was used for growth rate calculations as described in Sect. 2.3. For more details on the experiments involving organic vapors, see Lehtipalo *et al.*^42^ and Yan *et al.*^37^

#### 2.1.2 Particle number size distribution measurements

The particle size distribution in the CLOUD chamber was measured with a suite of instruments. The primary instrument for the measurement of the total particle size distribution below 10 nm was DMA-train.^53^ It utilizes six differential mobility analyzers (DMAs) in parallel at fixed voltages corresponding to different particle sizes. This provides significantly increased counting statistics and hence sensitivity to low number concentrations of particles compared to instruments which infer the size distribution by scanning procedures. The DMA-train is designed to minimize losses in the sub-10 nm range and uses particle detectors optimized for sub-3 nm particle detection. The instrument is described in more detail in Stolzenburg *et al.*^53^

We also used ion size distributions measured with the NAIS (Neutral cluster and Air Ion Spectrometer). The NAIS measures the size distribution of positively and negatively charged ions with mobility diameters between 0.8 and 42 nm.^54^

#### 2.1.3 Measurement of precursor vapor concentrations

The concentrations of sulfuric acid and HOMs were measured with a nitrate-ion based chemical ionization atmospheric pressure interface time-of-flight mass spectrometer (CI-APi-TOF).^21,55^ In the instrument, sulfuric acid and HOMs are charged by nitrate anions. After that, the charged ion clusters in the sample flow are focused in the APi part and detected in the TOF chamber based on their mass-to-charge ratio. The mass resolution of the instrument was about 4500 Th/Th, which allowed for assignment of the elemental formulae with satisfactory accuracy. In line with the earlier studies,^37,42^ compounds with a minimal carbon number of four were defined as HOM, which were further grouped to HOM monomers (4 ≤ carbon number ≤ 10) and HOM dimers (10 < carbon number ≤ 20) containing nitrogen (HOM_nit,mon_ and HOM_nit,dim_) and not containing nitrogen (HOM_non-nit,mon_ and HOM_non-nit,dim_). To quantify sulfuric acid and HOM concentrations, the system was calibrated with sulfuric acid and corrected for the mass-dependent transmission.^35,56^ The uncertainty in sulfuric acid and HOM concentrations is estimated to be approx. 40%, assuming a unit charging probability. However, the charging of HOMs can vary considerably, depending on the functionality and oxidation degree of HOMs^57^ and thus, the method gives a lower estimate of more volatile oxidation products (*e.g.* LVOCs).

The overall HOM volatility distribution can vary depending on NO_*x*_ concentration^37,39^ and thus the volatility distribution of HOM was not identical in all the studied experiments. More specifically, with an increasing amount of NO_*x*_, the concentration of HOM_non-nit,dim_ is largely reduced, while the concentrations of nitrogen-containing HOMs, including HOM_nit,mon_ and HOM_nit,dim_, are increased. From the perspective of HOM volatility, this causes a decrease in the concentrations of ELVOC compounds (saturation mass concentration *C*_mass,sat_ ≤ 10^−4.5^ μg m^−3^) and a slightly greater increase in the concentrations of LVOCs (10^−4.5^ μg m^−3^ < *C*_mass,sat_ ≤ 10^−0.5^ μg m^−3^).^37^

### 2.2 Cluster population simulations

#### 2.2.1 Cluster population model

We simulated the time-development of molecular cluster concentrations with a molecular-resolution cluster population model, similar to our earlier study.^33^ The simulations included one or two model substances, representing an inorganic compound and/or organic compounds. The cluster population was simulated from vapor monomers up to clusters or particles with a mobility diameter of 5.6–6 nm, including all possible cluster compositions. The largest simulated cluster contained 500 molecules in simulations with only one model compound and 200 or 240 molecules in the simulations with two model compounds. The model compounds are discussed more in Sect. 2.2.2.

In the model, the discrete General Dynamic Equation (GDE), including different processes where a cluster can be formed or lost, is numerically solved for each cluster composition i:1
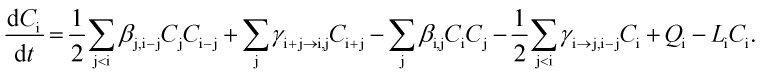
Here *C*_i_ is the concentration of cluster i and *β*_i,j_ is the collision rate coefficient between cluster i and cluster j. γ_i+j→i,j_ is the evaporation rate coefficient of cluster (i + j) to clusters i and j, which we allowed only for evaporation of vapor monomers. *Q*_i_ is the source rate, which was incorporated only for vapor monomers. *L*_i_ is the loss rate coefficient, describing the external sink of vapors and clusters. The GDEs were generated and solved with the ACDC (Atmospheric Cluster Dynamics Code) program.^58,59^

The collision rate coefficients *β*_i,j_ were calculated as hard-sphere collisions. In reality, the collision-coefficients may exceed the hard-sphere collision rates, due to van der Waal forces. This enhancement has been shown to be approximately a factor of 2 for sub-5 nm particles in sulfuric acid system.^36^ However, estimates for the magnitude of enhancement do not exist for organic systems, or for the mixed organic–inorganic systems. To be able to compare the variation of growth rate in different systems with inorganic and organic vapors, we chose not to include the enhancement for any of the studied systems. This simplification can cause simulated growth rates to be underestimated, at least in the simulations involving only inorganic compounds.

The evaporation rate coefficients of vapor monomers from different clusters were in most simulations calculated from the Kelvin equation:2
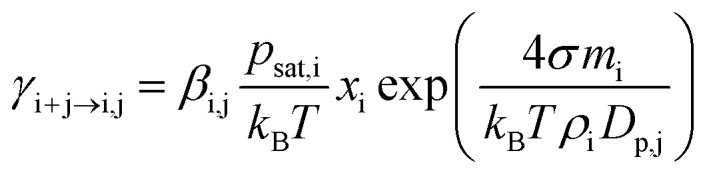
Here *β*_i,j_ is the collision rate coefficient between vapor compound i and cluster j. *p*_sat,i_ is the saturation vapor pressure of the compound i, *x*_i_ is the molar fraction of the compound i in cluster j, *σ* is the cluster surface tension, *ρ*_i_ is the liquid phase density of i, and *D*_p,j_ is the cluster diameter. *k*_B_ is the Boltzmann constant and *T* is the temperature. The Kelvin formula is the default approach for evaporation rates in single-particle condensational growth models, which have been applied to interpret observed growth rates.^12,21,23^

The loss coefficient *L*_i_ in eqn (1) was set to represent losses in the CLOUD chamber, entailing a size-dependent wall loss and a size-independent dilution loss.^60^

In addition to performing simulations with a model system, where the largest clusters contain hundreds of molecules, we also performed a few simulations with a clearly smaller model system, including only clusters containing 0–6 sulfuric acid as well as ammonia molecules. In these simulations, sulfuric acid and ammonia were treated as separate model compounds instead of a quasi-unary substance (see the next section). This allowed us to assess quantum-chemistry-based evaporation rates for the large model system,^61^ and to investigate the effects of simplifying a binary sulfuric acid–ammonia system to a quasi-unary system.

One should note that ion processes are not included in our simulations. The presence of ions can affect particle dynamics, by modifying the collision and evaporation rates.^14,49,62^ The ions of opposite polarity can also form neutral particles in ion–ion recombination, influencing the growing mode used for growth rate calculations.^15,63^ While a charge enhancement in growth has been observed in CLOUD experiments for the smallest particles (<2 nm) in the presence of sulfuric acid and ammonia,^14^ no charge enhancement was observed with organic vapors.^12^ As the current understanding of the effects of ions on the growth dynamics in inorganic–organic mixtures is limited, and the charged fractions of growing particles in the studied experiments are low,^36^ we chose to omit ions in our simulations.

#### 2.2.2 Model compounds

We performed simulations with one or two inorganic and organic compounds relevant for atmospheric new particle formation. The properties of the model substances are presented in Table 1.

**Table 1:** Model substances and their molecular mass (*m*), density (*ρ*), surface tension (*σ*), saturation vapor pressure (*p*_sat_), saturation number concentration (*C*_sat_) and saturation mass concentration (*C*_mass,sat_) at *T* = 278 K

Model substance	*m* (amu)	*ρ* (kg m^−3^)	*σ* (N m^−1^)	*p*_sat_ (Pa)	*C*_sat_ (cm^−3^)	*C*_mass,sat_ (μg m^−3^)
SA	142.6	1500.0	3.0 × 10^−2^	2.0 × 10^−9^	5.2 × 10^5^	1.2 × 10^−4^
LVOC	345.0	1500.0	3.0 × 10^−2^	1.0 × 10^−8^	2.6 × 10^6^	1.5 × 10^−3^
ELVOC	345.0	1500.0	3.0 × 10^−2^	1.0 × 10^−10^	2.6 × 10^4^	1.5 × 10^−5^

The inorganic compound represents a quasi-unary sulfuric acid–ammonia mixture (abbreviated here as SA). The mass of the compound was obtained by summing up the masses of one sulfuric acid molecule, 1/2 ammonia molecules and two water molecules. The resulting acid–base molar ratio of 2 : 1 is consistent with the experimental and theoretical studies suggesting that while cluster formation proceeds approximately by addition of acid–base pairs, the full neutralization of sulfuric acid by ammonia does not happen in small particles.^64,65^ The addition of water molecules is justified as sulfuric acid–ammonia particles are expected to contain some water.^66^ The value of *p*_sat_ for SA (2.0 × 10^−9^ Pa) was selected so that it resulted in test simulations in growth rates of the same order of magnitude with experimental values. It is also of the same order of magnitude with estimates of vapor pressure of sulfuric acid in partially neutralized solutions of sulfuric acid, ammonia and water.^18^

The organic compounds represent atmospheric oxidized organic species with two volatilities, corresponding to a LVOC and an ELVOC. Their properties (see Table 1) were selected to reasonably reflect the properties of oxidation products of monoterpenes based on previous CLOUD experiments.^38^ The values of *p*_sat_ used for LVOC and ELVOC (1.0 × 10^−8^ and 1.0 × 10^−10^ Pa) are consistent with the volatility basis set classification of organic compounds,^22^ although *p*_sat_ of ELVOC is close to the upper limit of the volatility bin commonly used for ELVOCs. It should be noted that representing oxidized organic compounds with only two components is a very simplified approach, as the chemical complexity of organics is not considered. Furthermore, in the following, our definitions for LVOC and ELVOC monomers and dimers are not the same as for HOM monomers and dimers. In the case of LVOC and ELVOC, the monomer refers to a molecule of the model compound and the dimer is a cluster composed of two monomers, while for HOMs, the division to monomers and dimers is based on the HOM chemical properties (see Sect. 2.1.3).

In most of the simulations, we calculated cluster evaporation rates from the Kelvin formula (eqn (2)) using *p*_sat_ values shown in Table 1. In reality, cluster evaporation rates are not expected to smoothly decrease with an increasing particle size as the Kelvin formula suggests but vary with the cluster composition. To study the effect of more realistic evaporation rates, we used quantum-chemistry-based evaporation rates for SA model compound in one simulation set. This was done as follows: (1) we retrieved from Besel *et al.*^61^ Gibbs free energies for the sulfuric acid–ammonia system, which had been obtained by applying a density functional theory (DFT) method for cluster structures and vibrational frequencies and the Domain based Local Pair Natural Orbital Coupled Cluster method (DLPNO-CCSD(T)) for single-point energy calculations. DLPNO-CCSD(T) is considered to be the best available quantum chemical method for atmospheric clusters.^67^ (2) We performed simulations with a cluster kinetics model treating sulfuric acid and ammonia as two separate model compounds. Sulfuric acid concentration was varied between 10^7^ and 10^8^ cm^−3^ and ammonia concentration was set to 43 ppt. Our model system included clusters containing 0–6 sulfuric acid and 0–6 ammonia molecules for which we calculated evaporation rates using Gibbs free energies by Besel *et al.*^61^ (3) From the model simulations, we determined the main cluster growth pathway (similar to Olenius *et al.*^68^). (4) We found the lowest evaporation rate (*i.e.*, the evaporation rate of the most stable cluster) for each set of clusters with a certain number of sulfuric acid molecules on the growth pathway and used those for 2–5-mers in our quasi-unary sulfuric acid–ammonia simulations. For larger clusters, we calculated evaporation rates from the Kelvin formula (eqn (2)), like in other simulations.

**Fig. 1:**
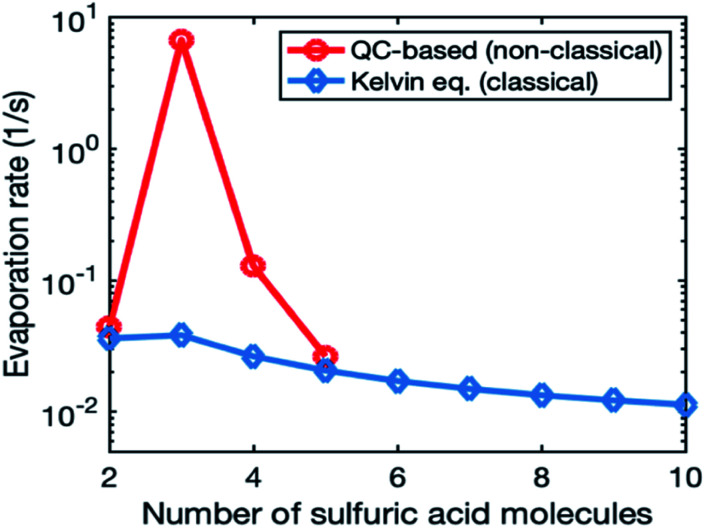
Evaporation rates obtained using quantum-chemistry (red line, circles) and Kelvin equation (blue line, diamonds) for 2–10-mers of a quasi-unary sulfuric acid–ammonia model compound.

Fig. 1 illustrates the evaporation rates obtained from quantum chemistry and the Kelvin equation for the smallest clusters (2–10-mers). In the quantum-chemistry-based evaporation rate profile, there is a clear maximum for trimer, while evaporation rates from the Kelvin equation decrease smoothly with an increasing number of molecules in the cluster. We do not use quantum-chemistry data for the organic system, as those data sets are currently very limited. This is because the relevant organic molecules have a high molecular mass and many possible rotamers, which makes quantum chemical calculations challenging.^67^

#### 2.2.3 Simulation sets

The simulation sets are described in Table 2. The simulation sets 1–3 were performed with one model compound and the simulation sets 4–5 with two model compounds. All the simulations were performed with constant vapor source rates, resulting in the steady-state vapor concentrations shown in the table. In the simulation set 2, the evaporation rates of the smallest clusters were obtained using quantum-chemistry data, while in other simulation sets, Kelvin equation was used for all the clusters. We will refer to these two simulation types as simulations with a classical evaporation rate profile and simulations with a non-classical evaporation rate profile. Temperature was set to 278 K in all the simulations.

**Table 2:** Description of the simulation sets. Vapor concentrations are steady-state values

Simulation set	Model compounds	Vapor concentrations (cm^−3^)	Method to retrieve evaporation rates
1	SA	*C*_SA_ = 8.0 × 10^6^, 2.0 × 10^7^, 4.7 × 10^7^, 1.1 × 10^8^	Kelvin eqn (classical evaporation rates)
2	SA	*C*_SA_ = 2.0 × 10^7^, 4.7 × 10^7^, 1.1 × 10^8^	QC data and Kelvin eqn (non-classical evaporation rates)
3	LVOC	*C*_LVOC_ = 5.0 × 10^7^, 1 × 10^8^	Kelvin eqn (classical evaporation rates)
4	LVOC	*C*_LVOC_ = 5.0 × 10^7^, 1 × 10^8^	Kelvin eqn (classical evaporation rates)
	ELVOC	*C*_ELVOC_ = 1.0 × 10^7^	
5	LVOC	*C*_LVOC_ = 2.0 × 10^7^, 5.0 × 10^7^, 1 × 10^8^	Kelvin eqn (classical evaporation rates)
	SA	*C*_SA_ = 8.0 × 10^6^	

These simulations were chosen to elucidate how the presence of one or two condensable vapors with different evaporation rates and concentrations influences (1) the size-dependency of observed growth rates, and (2) the threshold size above which stochastics effects can be neglected. While cluster population simulations allow us to consider the impacts of population dynamics, including stochastic effects, on particle growth, the main drawback of this approach is high computational costs. For that reason, the number of model components was limited to two and the largest simulated particle size did not exceed 6 nm. For SA, we used similar vapor concentrations in the simulations to those observed in the corresponding experiments. However, for organic compounds, we did not exactly match the simulated vapor concentrations with the measured values, due to the limited number of different organic vapors in the simulations and high uncertainties in their evaporation rates. Instead, the simulated organic concentrations correspond to the approximate range of vapor concentrations in the studied experiments.

### 2.3 Determining particle growth rates

We determined particle growth rates from experimental and simulation data by using an appearance time method,^69^ which is a common approach to derive observed growth rates in the sub-5 nm size range from chamber experiments. The method is based on determining the times *t*_app,i_ when particle concentration in size bin with mean diameter *D*_p,i_ reaches 50% of the total increase in the concentration. For a discussion on the differences between this and other approaches used to determine particle growth rate, see Dada *et al.*^70^

From experimental data we determined *t*_app,i_ by fitting a Sigmoid function to measured signal in each size channel. Then we determined particle growth rate and its uncertainty by using a Monte Carlo simulation.^70^ We obtained the uncertainty of *t*_app,i_ from the uncertainty of the Sigmoid fit and the uncertainty of *D*_p_,_i_ from the instrumental parameters and assumed that these uncertainties are normally distributed. Then we reproduced 10 000 data sets by randomly selecting values for *t*_app,i_ and *D*_p_,_i_ from their estimated distributions and calculated growth rate from each data set using a total least squares method. We obtained the final value of growth rate as the median value of all calculated growth rates and its uncertainty as their standard deviation. We determined the growth rate for two size ranges: *D*_p_ = 1–3 nm (referred to as GR_1–3_) and *D*_p_ = 3–7 nm (GR_3–7_). We used both DMA-train and NAIS data to calculate growth rate in pure organic experiments and in mixed inorganic–organic experiments. In the experiments involving only sulfuric acid and ammonia, we used only DMA train data because in these experiments charged particles, measured with NAIS, grew faster than the total particle population, as also shown by Stolzenburg *et al.*^36^

To treat the simulation data similarly to experimental data, we divided the simulated clusters in linearly spaced size bins of a width of 0.1 nm, based on their mobility diameter. Because the simulated concentrations do not fluctuate similarly to measurement data, we did not use Sigmoid fits but determined *t*_app,i_ for each size bin directly from simulated concentrations. Then we used a least square fitting to determine growth rate for two size ranges: *D*_p_ = 1–3 nm (GR_1–3_) and *D*_p_ = 3–5 nm (GR_3–5_). The larger size range extends only to 5 nm, because we wanted to be sure that boundary effects, which can be significant close to the upper limit of the simulation system, do not distort our results. In the simulations including SA and LVOC, the upper limit was set to 4.5 nm for the same reason. To assess the mechanisms driving particle growth in our simulations, we also investigated particle fluxes due to different collisions and evaporations past selected threshold sizes. We chose not to convert these to flux equivalent particle growth rates (see Kontkanen *et al.*^43^), as the resulting growth rates would not correspond to any observed particle growth rate and they would contain uncertainties related to the conversion.

### 2.4 Metric for determining the threshold size for stochastic effects

We applied a metric introduced by Olenius *et al.*^8^ to investigate the importance of stochastic effects in particle growth in different systems. The metric is based on studying the ratio of the absolute values of the second and first derivative of the particle size distribution:3
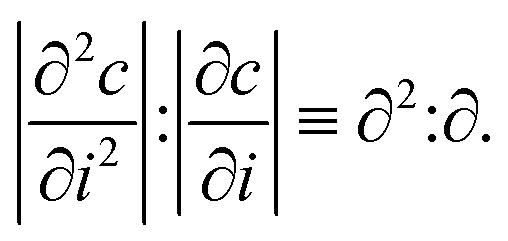
Here *c*(*i*,*t*) is the size distribution function and *i* is the size of the particle as a number of molecules. The idea behind the metric can be understood by considering that the representation of condensational growth in single-particle and aerosol dynamics models is based on the continuous GDE, which is derived from the explicit discrete GDE by approximating particle size as a continuous variable. This leads to a condensational growth flux equation that is analogous to the convection–diffusion equation, with a first-order drift term (∝ ∂*c*/∂*i*) corresponding to the driving force of condensation (the difference between the collision and evaporation rate constants) and a second-order diffusion term (∝ ∂^2^*c*/∂*i*^2^) corresponding to stochastic molecular collisions and evaporations. For larger aerosol particles the latter term is generally omitted but it should be included if it is comparable to the first-order term, that is, ∂^2^:∂ ≫ 0.

Olenius *et al.*^8^ showed that ∂^2^:∂ can be used to assess the size regime above which stochastic effects become negligible. Namely, at the particle size *D*_p,th_ where ∂^2^:∂ becomes very small (of the order of <5%), the relative difference between the growth rate due to only deterministic condensation and the growth rate including stochastic effects becomes negligible. Thus, *D*_p,th_ can be used as a threshold size above which the standard single-particle approach to interpret condensational growth (see Sect. 2.5) becomes valid. We determined *D*_p,th_ both from experimental data and simulations using the size distributions under steady-state condition, as the experimental data fluctuate less at the final state. We left out the experiments where the size distribution fluctuated too much, or simulated cluster concentrations became too low, for us to be able to determine *D*_p,th_ reliably.

### 2.5 Particle growth rate from deterministic condensation flux

To study the uncertainty of interpreting observed particle growth using a deterministic condensation model, which does not include stochastic effects or coagulational growth, we calculated particle growth rate based on deterministic condensation flux on a single particle according to:^8^4
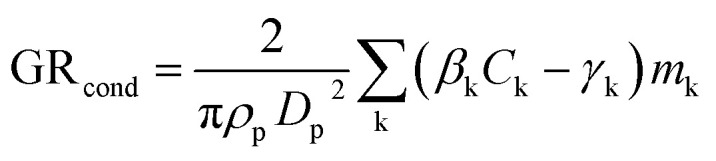
Here *D*_p_ and *ρ*_p_ are the diameter and density of the particle, *β*_k_ is the collision rate coefficient between vapor molecule k and the particle, *γ*_k_ is the evaporation rate of vapor molecule k from the particle, *C*_k_ is the concentration of vapor k and *m*_k_ is its molecular mass.

We calculated condensation growth rate from eqn (4) for 1–5 nm particles, considering the condensation of SA model compound at *C*_SA_ = 1.0 × 10^7^, 4.7 × 10^7^ and 1.1 × 10^8^ cm^−3^. We calculated *β*_k_ as hard-sphere collisions and obtained *γ*_k_ from Kelvin equation (eqn (2)).

## 3. Results

### 3.1 Size-dependence of growth rate in experiments

**Fig. 2:**
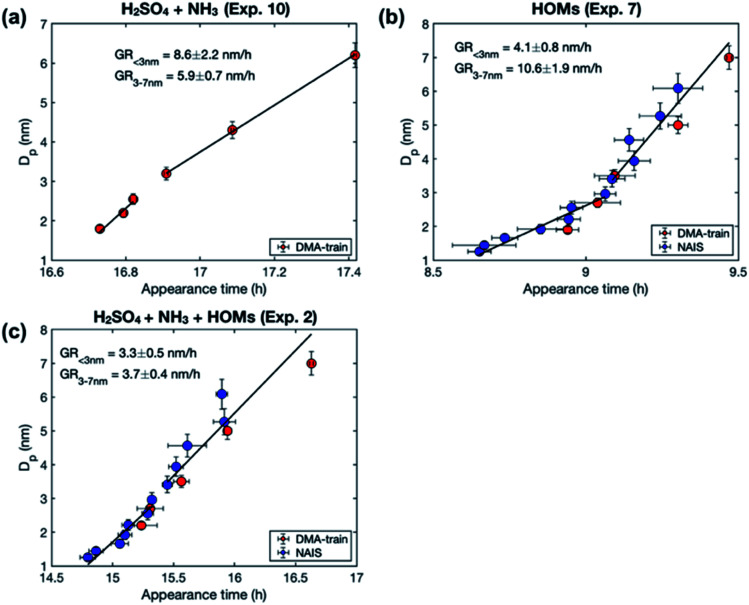
Examples of the size-dependency of growth rate in experiments involving different precursor vapors: (a) sulfuric acid (H_2_SO_4_) and ammonia (NH_3_). (b) HOMs, and (c) the mixture of H_2_SO_4_, NH_3_ and HOMs. The markers show particle diameters (*D*_p_) as a function of the corresponding appearance times from different instruments and the error bars show their uncertainty. Solid black lines show the linear fits to the data points in two size ranges (1–3 and 3–7 nm). Growth rates (GR), obtained as slopes of the fits, are shown in the figures. For the vapor concentrations in each experiment, see Table 3.

We observe different size-dependency of the growth rate in different sets of experiments, depending on the precursor vapors present in the chamber (Fig. 2). Table 3 shows the particle growth rates in two studied size ranges and their ratio, as well as the concentrations of sulfuric acid and HOMs for each experiment (see Table S1† for more details). In the experiments with only sulfuric acid and ammonia, the growth rate decreases with size: the ratio between GR_3–7_ and GR_1–3_ is 0.7–0.8. A similar size-dependence of the growth rate has been reported previously for the same experiments.^36^ It can be explained by two mechanisms: (1) in collision-limited condensation, the growth rate decreases with size because the vapor molecule size relative to particle size decreases,^25^ and (2) collision enhancement due to van der Waals forces is reduced, when particle size increases.^36^

**Table 3:** Summary of the experimental data, including growth rates (GRs) in two size ranges (subscripts refer to size range limits in nm) and their ratio, the steady state concentrations of sulfuric acid (H_2_SO_4_) and HOM monomers (HOM_mon_) and HOM dimers (HOM_dim_), the ratio between the total concentrations of HOMs not containing nitrogen (HOM_non-nit,tot_) and HOMs containing nitrogen (HOM_nit,tot_), and the threshold size above which the stochastic effects in particle growth can be neglected (*D*_p,th_). More details on precursor gas concentrations in these experiments are shown in Table S1

Exp. no.	GR_1–3_ (nm h^−1^)	GR_3–7_ (nm h^−1^)	GR_3–7_/GR_1–3_	H_2_SO_4_ (cm^−3^)	HOM_mon_ (cm^−3^)	HOM_dim_ (cm^−3^)	HOM_non-nit,tot_/HOM_nit,tot_	*D*_p,th_ (nm)
1	4.2 ± 0.8	3.5 ± 0.6	0.8	7.8 × 10^6^	2.5 × 10^7^	3.2 × 10^6^	1.0	2.9
2	3.3 ± 0.5	3.7 ± 0.4	1.1	7.9 × 10^6^	2.2 × 10^7^	2.9 × 10^6^	1.1	3.0
3	8.4 ± 2.8	3.5 ± 0.4	0.4	9.7 × 10^6^	2.2 × 10^7^	2.8 × 10^6^	1.0	2.9
4	4.4 ± 0.9	3.6 ± 0.4	0.8	8.3 × 10^6^	1.5 × 10^7^	3.0 × 10^6^	1.0	3.0
5	9.8 ± 9.0	20.8 ± 5.7	2.1	—	2.6 × 10^7^	7.8 × 10^6^	1.7	3.5
6	4.5 ± 0.8	13.3 ± 3.6	3.0	—	1.9 × 10^7^	5.0 × 10^6^	3.4	3.5
7	4.1 ± 0.8	10.6 ± 1.9	2.6	—	1.9 × 10^7^	5.1 × 10^6^	3.5	3.5
8	3.4 ± 0.6	12.2 ± 4.2	3.6	—	1.8 × 10^7^	4.8 × 10^6^	3.5	—
9	3.5 ± 1.0	2.6 ± 0.3	0.7	2.0 × 10^7^	—	—	—	2.6
10	8.6 ± 2.2	5.9 ± 0.7	0.7	4.7 × 10^7^	—	—	—	2.5
11	17.0 ± 5.5	14.3 ± 1.7	0.8	1.1 × 10^8^	—	—	—	—

In the experiments with only organic vapors in the chamber, growth rate increases with size: the ratio between GR_3–7_ and GR_1–3_ is 2.1–3.6. The increasing growth rate of sub-10 nm particles with increasing size has been previously observed in CLOUD experiments involving only biogenic vapors.^12,38^ It has been explained by decreasing Kelvin effect with increasing size, allowing a nano-Köhler type activation, where organic vapors with higher volatility can condense on particles when they grow.

In the experiments involving both sulfuric acid and organic vapors, one could also expect to see an increasing growth rate with the increasing size. However, in the experiments studied here, the growth rate either decreases with size or stays almost constant, the ratio between GR_3–7_ and GR_1–3_ varying between 0.4 and 1.1. The behavior of growth rate could be connected to the presence of sulfuric acid or to the volatility distribution of HOMs. As shown in Table 3, in the experiments involving both sulfuric acid and HOMs (Exp. 1–4), the ratio between the total concentrations of HOMs not containing nitrogen (HOM_non-nit,tot_) and nitrogen containing HOMs (HOM_nit,tot_) is ∼1.0, while in the experiments with only HOMs (Exp. 5–8), this ratio is 1.7–3.5. This is mainly due to the difference in the ratio of NO_*x*_ concentration to the monoterpene concentration (NO_*x*_/VOC) between the experiments – a lower NO_*x*_/VOC leads to a higher HOM_non-nit,tot_/HOM_nit,tot_. With respect to the organic volatility distribution, low NO_*x*_/VOC leads to a higher ELVOC concentration and a lower LVOC concentration, compared to high NO_*x*_/VOC conditions.^37,39^ We will discuss the possible explanations for the size-dependency of growth rate in these experiments in Sect. 3.3, based on our modeling results. Generally, the observation that the growth rate does not increase with size in these experiments indicates that nano-Köhler type behavior, with clearly increasing growth rate with size, occurs only under specific constraints. This is consistent with our previous simulation results, suggesting that nano-Köhler type activation occurs in a system involving sulfuric acid and organics only when the saturation ratio of organic vapor and the ratio between organic and sulfuric acid concentrations are in a suitable range.^33^

### 3.2 Growth rate in simulations with sulfuric acid

Growth rate was found to decrease with size in the simulations involving only SA (Table 4), qualitatively similar to the experiments involving sulfuric acid and ammonia. The ratio between GR_3–5_ and GR_1–3_ is 0.8–0.9, which is close to the ratio observed in the experiments (see Table 3). Slightly higher values of the ratio in the simulations compared to the experiments (*i.e.* less strongly decreasing growth rate) could result from our simulations being quasi-unary, and not considering van der Waals forces.^36^ Another possible reason for the difference is that the upper limit of the larger size range for which growth rate is calculated extends to higher sizes (7 nm) in experiments than in the simulations (5 nm).

**Table 4:** Summary of the simulation results, including the steady state vapor concentrations of SA, LVOC and ELVOC, growth rates (GRs) in different size ranges (subscripts refer to size range limits in nm), and the threshold size above which stochastic effects in particle growth can be neglected (*D*_p,th_)

Simulation no.	Model compounds	*C*_SA_ (cm^−3^)	*C*_LVOC_ (cm^−3^)	*C*_ELVOC_ (cm^−3^)	GR_1–3_ (nm h^−1^)	GR_3–5_ (nm h^−1^)	GR_3–5_/GR_1–3_	*D*_p,th_ (nm)
1	SA	8.0 × 10^6^	—	—	0.8	0.6	0.8	3.6
2	SA	2.0 × 10^7^	—	—	3.2	2.5	0.8	2.8
3	SA	4.7 × 10^7^	—	—	21.4	19.5	0.9	2.8
4	SA	1.1 × 10^8^	—	—	68.3	63.4	0.9	2.8
5	SA^a^	2.0 × 10^7^	—	—	2.0	1.8	0.9	2.9
6	SA^a^	4.7 × 10^7^	—	—	10.7	9.0	0.8	2.6
7	SA^a^	1.1 × 10^8^	—	—	47.8	44.6	0.9	2.7
8	LVOC	—	5.0 × 10^7^	—	3.5	2.0	0.6	—
9	LVOC	—	1.0 × 10^8^	—	3.5	4.9	1.4	—
10	LVOC, ELVOC	—	5.0 × 10^7^	1.0 × 10^7^	2.1	4.5	2.1	3.7
11	LVOC, ELVOC	—	1.0 × 10^8^	1.0 × 10^7^	2.6	10.1	3.9	3.8
12	LVOC, SA	8.0 × 10^6^	2.0 × 10^7^	—	1.0	1.9	1.9	3.6
13	LVOC, SA	8.0 × 10^6^	5.0 × 10^7^	—	1.2	4.1	3.4	3.7
14	LVOC, SA	8.0 × 10^6^	1.0 × 10^8^	—	1.7	9.9	5.8	3.6

a Non-classical evaporation rates.

**Fig. 3:**
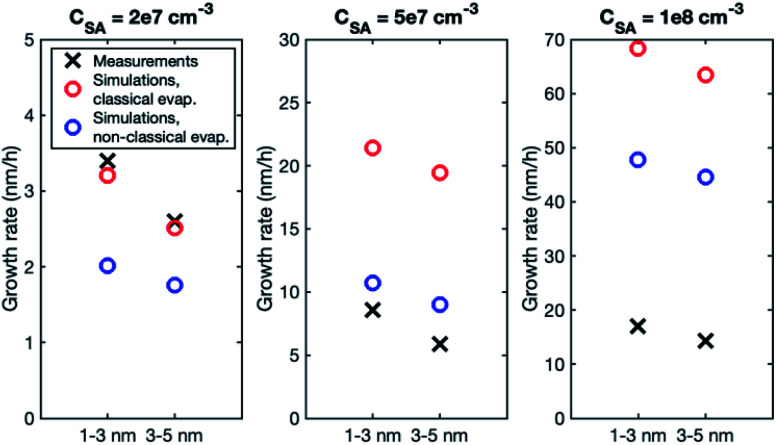
Particle growth rate in two size ranges in measurements (black crosses) and in simulations with a classical evaporation rate profile (red circles) and a nonclassical evaporation rate profile (blue circles). Sulfuric acid concentration in each case is shown above the figure.

Although the size dependence of growth rate is rather similar in simulations and experiments, the absolute values of growth rate differ, especially at higher vapor concentrations. Fig. 3 shows the growth rates determined from simulations with classical and nonclassical evaporation rate profiles and the corresponding experimental values. At *C*_SA_ = 2 × 10^7^ cm^−3^, experimental growth rates are only slightly higher than simulated values, with the difference being larger for a nonclassical evaporation rate profile. However, at higher vapor concentrations, simulated growth rates become clearly higher than experimental growth rates. This is obvious especially in the simulations with a classical evaporation rate profile for which GR_1–3_ is by a factor of 2.5 and 4.0 higher than experimental values at *C*_SA_ = 5 × 10^7^ cm^−3^ and *C*_SA_ = 1 × 10^8^ cm^−3^, respectively. Thus, in the simulations, the growth rate increases with increasing sulfuric acid concentration clearly more than in the experiments.

**Fig. 4:**
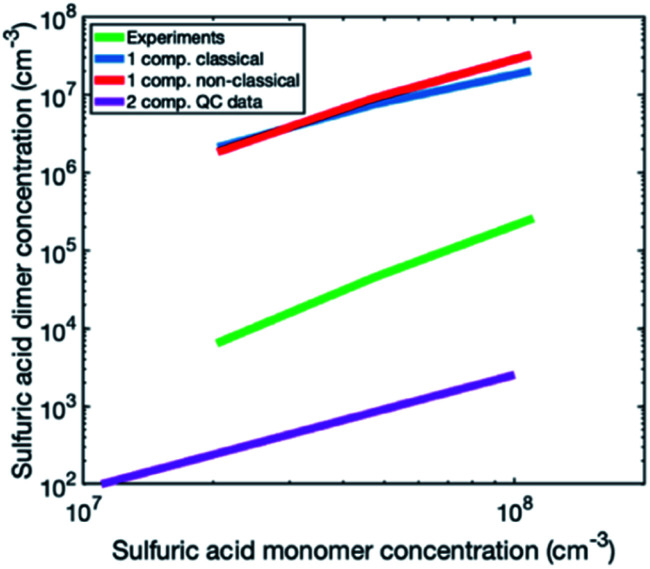
Steady-state sulfuric acid monomer and dimer concentrations in experiments (green line) and in different types of simulations: one-component simulations with a classical evaporation rate profile (blue line), one-component simulations with a nonclassical evaporation rate profile (red line), and two-component simulations using quantum chemistry data for all the clusters (purple line). In experiments and two-component simulations, sulfuric acid monomer and dimer concentrations include sulfuric acid monomers and dimers with 0–*n* ammonia molecules.

To investigate the reason for the differences between simulations and experiments, we compared sulfuric acid dimer concentrations in experiments and different simulations (Fig. 4): one-component simulations with a classical evaporation rate profile, one-component simulations with a nonclassical evaporation rate profile, and two-component simulations, where sulfuric acid and ammonia are separate model compounds and quantum chemical data is used for all the simulated clusters. In the one-component simulations, sulfuric acid dimer concentrations at certain monomer concentrations are similar with the two evaporation rate profiles and clearly higher (by two orders of magnitude) than dimer concentrations in the experimental data. However, in the two-component simulations, dimer concentrations are lower than in the experimental data (by one to two orders of magnitude). This comparison suggests that using one-component simulation system for sulfuric acid and ammonia results in unrealistically high dimer concentrations. Previously, one-component cluster population simulations have been used for describing particle formation involving sulfuric acid and dimethylamine and the measured and simulated cluster concentrations have been found to agree reasonably well.^71^ The better agreement for sulfuric acid–dimethylamine system can be explained by dimethylamine being a significantly stronger base than ammonia, making the first cluster consisting of one sulfuric acid molecule and one dimethylamine much more stable than the corresponding cluster with ammonia.^72^ The large difference between the dimer concentrations in the experiments and in the two-component simulations can result from the tendency of the used quantum chemistry method to slightly underestimate the cluster stability,^61^ illustrating the need to improve these methods (see Elm *et al.*^67^).

**Fig. 5:**
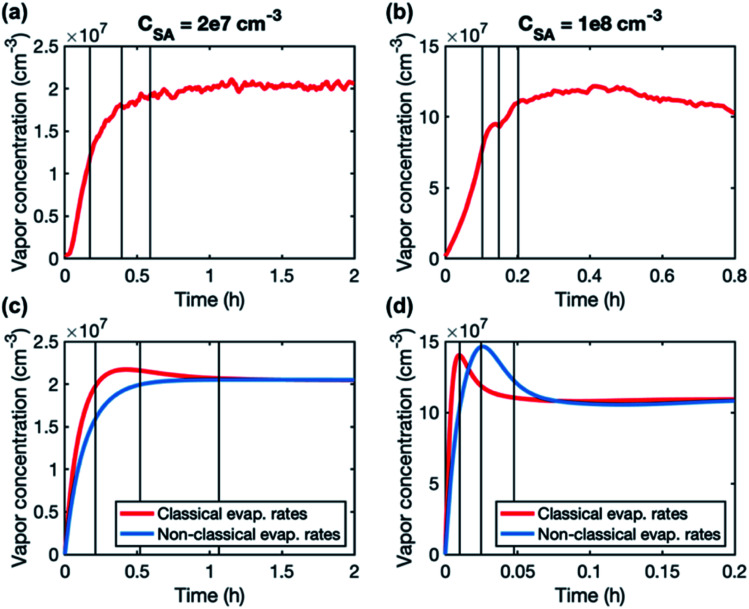
Time-evolution of sulfuric acid concentrations in two experiments (a and b) and in the corresponding simulations (c and d). The black vertical lines show appearance times of 1.5, 2.5 and 4 nm particles. In the simulation figures (c and d), the shown appearance times correspond to the simulations with a classical evaporation rate profile.

A higher contribution of clusters to particle growth can explain clearly higher growth rates in simulations than in experiments at high sulfuric acid concentrations. The time-dependence of vapor concentrations also suggests higher cluster concentrations in the simulations (Fig. 5). At *C*_SA_ = 2 × 10^7^ cm^−3^, simulated and measured vapor concentrations behave rather similarly, increasing until a steady state value is reached. However, at *C*_SA_ = 1 × 10^8^ cm^−3^, simulated vapor concentration has a clear maximum that is not observed in the experiments, and which can be explained by the interaction between vapor monomers and clusters. In the beginning of the simulation, cluster concentrations are still low and thus vapor concentration can reach its peak value. Then, the cluster concentrations increase, and the vapor is reduced due to collisions with the clusters, until a steady state is reached. A similar behavior is not observed in the experiments, because the concentrations of clusters are low and thus they do not act as a significant sink for the vapor. The time-dependent variation in the vapor concentration can also influence the size-dependence of growth rate.^33^ As shown in Fig. 5, the measured vapor concentration increases between the appearance times of 1.5 nm and 4 nm particles by a factor of ∼1.5. However, in the simulation with high SA concentration, vapor concentration decreases between these appearance times, which may enhance the reduction of growth rate with size.

**Fig. 6:**
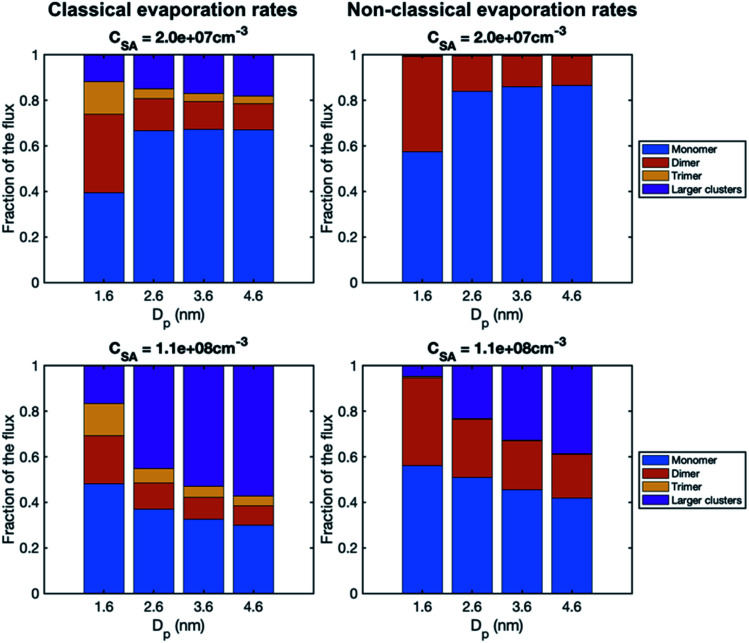
Contribution of vapor monomers and clusters to the particle flux past different threshold sizes in simulations with SA at two different concentrations (*C*_SA_ = 2.0 × 10^7^ and 1.1 × 10^8^ cm^−3^). The values are at the steady state. The corresponding absolute fluxes are shown in Fig. S1.†

To study the growth mechanisms in our simulations in more detail, we investigated the contributions of vapor monomers and clusters to particle fluxes past selected threshold sizes (Fig. 6). In line with the above, at low SA concentration, vapor monomer dominates the growth at sizes larger than 1.6 nm. Additionally, in the simulation with a classical evaporation rate profile, larger clusters have a non-negligible contribution to the growth at all sizes, while with a nonclassical evaporation rate profile, only dimer contributes to the growth besides vapor monomer. At high vapor concentrations, the difference between the classical and nonclassical cases becomes clearer: for the former, clusters larger than trimer dominate the particle growth at sizes above 1.6 nm while for the latter the growth mainly proceeds through monomers and dimers. Thus, the classical evaporation rate profile results in a more significant contribution of clusters to growth, leading to very high growth rates at high vapor concentrations.

Overall, in our simulations with a quasi-unary sulfuric acid–ammonia mixture, growth rate decreases with size, which is qualitatively similar to experimental observations, and can be explained by the decreasing vapor molecule size compared to particle size. Previous studies have found clusters to have a negligible contribution to particle growth in CLOUD experiments involving sulfuric acid and ammonia,^36,73^ which is consistent with our experimental results, but different from the simulation results for high vapor concentrations. The overestimation of the role of clusters in our simulations can result from using one-component model system and incorrect evaporation rate profiles, especially in the case of classical rates. This has implications also on the application of single-particle condensation models with classical evaporation rates: even if observed growth rates can be reproduced with such models, the modeled condensational growth rates are incorrect (even in the classical thermodynamics framework), as the classical rates lead to a significant contribution of clusters to particle growth. That is, applying Kelvin-based classical thermodynamics and assuming only vapor condensation is inconsistent at elevated vapor concentrations.

### 3.3 Growth rate in simulations involving organics

**Fig. 7:**
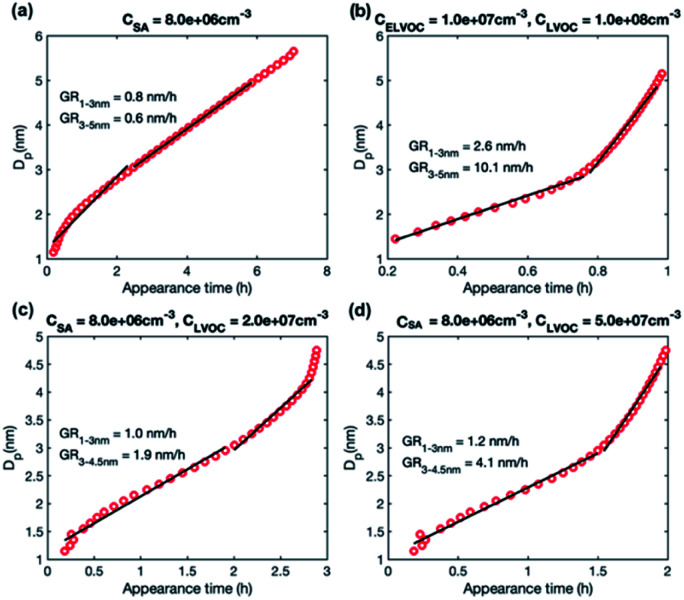
Particle diameter plotted as a function of appearance time in simulations with different model compounds: (a) SA at *C*_SA_ = 8.0 × 10^6^ cm^−3^, (b) LVOC and ELVOC at *C*_LVOC_ = 1 × 10^8^ and *C*_ELVOC_ = 1 × 10^7^ cm^−3^ (c) SA and LVOC at *C*_SA_ = 8 × 10^6^ and *C*_LVOC_ = 2 × 10^7^ cm^−3^, (d) SA and LVOC at *C*_SA_ = 8 × 10^6^ and *C*_LVOC_ = 5 × 10^7^ cm^−3^. Growth rate (GR) values determined for two size ranges with a linear fit are shown in the figures, while growth rates obtained for each size as a slope of the curve are shown in Fig. S2.†

We studied growth rates in simulations including (1) only LVOC, (2) ELVOC and LVOC, and (3) SA and LVOC. The simulation results are summarized in Table 4. Fig. 7 shows the appearance times of different sized particles in a few selected simulations and Fig. S2† the corresponding behavior of growth rate as a function of particle size.

In the simulations with only LVOC, the size-dependence of growth rate depends on LVOC concentration (shown only in Table 4). At *C*_LVOC_ = 5 × 10^7^ cm^−3^, growth rate decreases with size while at *C*_LVOC_ = 1 × 10^8^ cm^−3^, growth rate increases with size. The behavior of growth rate can be understood by studying the contribution of vapor monomers and clusters to particle flux (Fig. S3†). In both simulations, vapor monomer starts to dominate the growth flux above 3 nm, when its net flux becomes positive. When looking at the absolute values of the flux, at higher LVOC concentration, the partial flux due to vapor monomer barely decreases with the increasing particle size above 3 nm, despite the coagulation losses. This is due to the enhanced condensation of LVOC at these sizes, leading to the increase of growth rate with the increasing particle size.

**Fig. 8:**
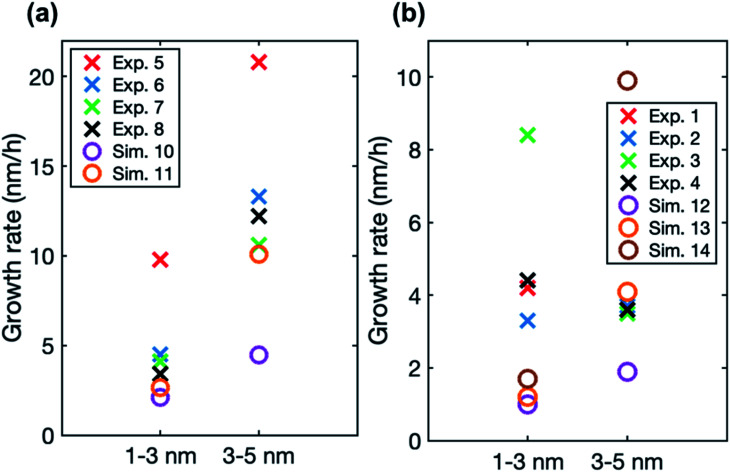
Particle growth rate in two size ranges in experiments (crosses) and in simulations (circles) with (a) LVOC and ELVOC and (b) LVOC and sulfuric acid. For the vapor concentrations in each case, see Tables 3 and 4.

In the simulations involving both LVOC and ELVOC, growth rate increases with size (see Table 4 and Fig. S2†), the ratio between GR_3–5_ and GR_1–3_ being 2.1 and 3.9 in the two simulations. This is qualitatively similar to the experiments involving only organics. Fig. 8a shows the comparison between growth rates in the experiments and simulations involving only organic vapors. In the simulation with *C*_ELVOC_ = 1 × 10^7^ cm^−3^ and *C*_LVOC_ = 1 × 10^8^ cm^−3^ (Sim. 11), growth rate is within a factor of 2 of most of the experimental values in both studied size ranges. In one of the experiments (Exp. 5), growth rates are clearly higher at both sizes, likely due to higher HOM concentrations (see Table 3). Considering the simplifications of our model simulations, such as including only two organic compounds and using classical evaporation rates, the qualitative agreement between the simulated and experimental growth rates is good. In the simulations, the total concentration of organic compounds is higher than in the experiments with similar growth rate values, but the CI-APi-TOF is known to underestimate measured LVOC concentrations,^12,36^ which could partly explain this.

**Fig. 9:**
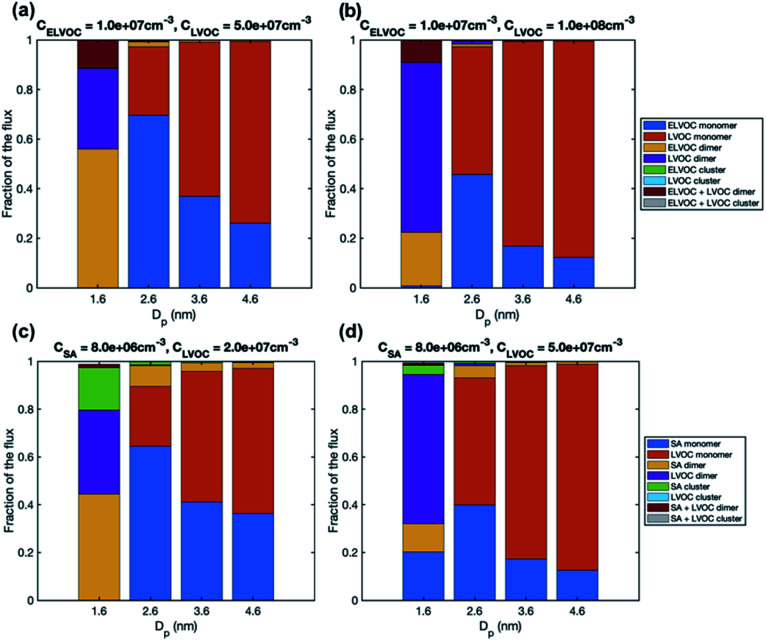
Contribution of vapor monomers and clusters to the particle flux past different threshold sizes in simulations with (a) LVOC and ELVOC at *C*_ELVOC_ = 10^7^ cm^−3^ and *C*_LVOC_ = 5 × 10^7^ cm^−3^, (b) LVOC and ELVOC at *C*_ELVOC_ = 10^7^ cm^−3^ and *C*_LVOC_ = 10^8^ cm^−3^, (c) SA and LVOC at *C*_SA_ = 8 × 10^6^ and *C*_LVOC_ = 2 × 10^7^ cm^−3^, (d) SA and LVOC at *C*_SA_ = 8 × 10^6^ and *C*_LVOC_ = 5 × 10^7^ cm^−3^. The values are at the steady state. The corresponding absolute fluxes are shown in Fig. S4.†

The increase of growth rate with size in the simulations with LVOC and ELVOC can be explained by the increasing contribution of more abundant LVOC to growth with the increasing size (Fig. 9 and S4†). In both simulations with LVOC and ELVOC, the net flux of vapor monomer is negative at the smallest size (1.6 nm), due to its high evaporation flux, and thus the growth flux is attributed mainly to ELVOC and LVOC dimers. Above that, the growth flux is caused by ELVOC and LVOC monomers, the contribution of LVOC monomer increasing with the increasing size, until at 4.6 nm it clearly dominates the flux in both simulations. The absolute values of the fluxes (Fig. S4†) show that the net flux due to LVOC monomer does not significantly decrease with the increasing particle size, despite coagulation losses, which can be explained by its increasing condensation efficiency.

In the simulations with LVOC and SA (*C*_SA_ = 8 × 10^6^ in all these simulations), growth rate increases with size, but the strength of the increase depends strongly on the LVOC concentration (see Table 4). At *C*_LVOC_ = 2 × 10^7^ cm^−3^, the ratio between GR_3–5_ and GR_1–3_ is 1.9. At higher LVOC concentrations, the increase of growth rate with size is more pronounced: the ratio between GR_3–5_ and GR_1–3_ is 3.4 and 5.8 (see Fig. 7 and S2†). Fig. 8b shows the comparison between growth rates in these simulations and in the experiments involving sulfuric acid and organic vapors. In the simulation with *C*_LVOC_ = 2 × 10^7^ cm^−3^ (Sim. 12), simulated GRs are lower than the experimental values in both size ranges. With higher LVOC concentrations (Sim. 13 and 14), GR_1–3_ is lower and GR_3–5_ higher in simulations than the corresponding values in the experiments.

The size dependence of growth rate in the simulations with SA and LVOC can be understood by studying particle fluxes (Fig. 9 and S4†). At the smallest size, the net flux of LVOC and SA monomers is negative, and the growth flux is attributed mainly to the dimers of SA and LVOC. Above 1.6 nm, the flux in both simulations is caused by SA and LVOC monomers, the contribution of LVOC monomer increasing with the increasing size and LVOC concentration. When studying the absolute fluxes (Fig. S4†), all the partial fluxes can be observed to clearly decrease with increasing particle size at *C*_LVOC_ = 2 × 10^7^ cm^−3^, due to coagulation losses. However, at higher LVOC concentration, the decrease of fluxes with size, especially that caused by LVOC monomer, is significantly smaller, due to more efficient condensation of LVOC. This corresponds to nano-Köhler type activation of particles to growth by an organic vapor^32^ and illustrates how the activation type behavior occurs only at specific vapor concentrations. Although LVOC significantly contributes to particle growth also in the simulation with *C*_LVOC_ = 2 × 10^7^ cm^−3^, a stronger increase in growth rate with size requires a higher LVOC concentration.

Our simulations suggest that the increase of growth rate with size is largely governed by LVOC concentration. The particle growth below 3 nm is almost solely due to SA and/or ELVOC (because they have low enough volatility), which is illustrated by GR_1–3_ being similar in the simulations with different LVOC concentrations (see Table 4). However, above 3 nm, the contribution of LVOC is important: increasing LVOC concentration enhances GR_3–5_ significantly in our simulations. Table 3 shows that in most experiments involving organics with or without sulfuric acid, GR_1–3_ are rather close to each other (∼3–4 nm h^−1^), while GR_3–7_ is clearly lower in the experiments with both sulfuric acid and organics. This could be explained by lower LVOC concentration, but as discussed in Sect. 3.1, the fractions of different HOMs in the two types of experiments indicate even higher LVOC concentration in the mixed organic–inorganic experiments compared to pure organic experiments. Another potential explanation to the different size-dependency in the experiments could be the difference in the time-dependence of vapor concentrations, but the vapor concentrations were kept constant in all the experiments involving organics. Thus, the reason for the growth rate not increasing with size in the experiments involving both sulfuric acid and organic vapors remains unresolved and needs further investigation.

### 3.4 Importance of stochastic effects in particle growth

To investigate the importance of stochastic effects in particle growth, we determined the threshold sizes *D*_p,th_ above which metric ∂^2^:∂ becomes less than 0.05 in different experiments (Table 3). In practice, these effects are mainly driven by evaporation and thus they extend to sizes at which evaporation rates are comparable to vapor collision rates. In terms of particle diameter, these sizes can be larger for larger molecules, as larger molecules result in a larger diameter for a given number of molecules in the particle.

*D*_p,th_ is lowest (2.5–2.6 nm) in the experiments involving only sulfuric acid and ammonia. This is expected because in these experiments vapor molecules are smallest, and evaporation can be assumed to be least significant. The result is consistent with Stolzenburg *et al.*^36^ who concluded that evaporation is negligible for particles larger than 2 nm in these experiments. In the experiments involving both sulfuric acid and HOMs, *D*_p,th_ is 2.9–3.0 nm. This suggests more significant evaporation than in the experiments with sulfuric acid and ammonia, due to the presence of HOMs with varying volatilities. As expected, *D*_p,th_ is highest (3.5 nm) in the experiments with only HOMs, which are larger than sulfuric acid molecules and have a wide range of volatilities.

To assess the sensitivity of the obtained *D*_p,th_ to the assumed vapor molecule size, which is needed for extracting ∂^2^:∂ from the size distribution function, we tested determining *D*_p,th_ using the mass of SA in the equation for ∂^2^:∂ (see eqn (11) in Olenius *et al.*^8^) for all the experiments. In this case, *D*_p,th_ is reduced by ∼0.3 nm in the experiments with sulfuric acid and HOMs and by ∼0.5 nm in the presence of only HOMs. Still, the values of *D*_p,th_ are slightly higher in the experiments involving organics than with only sulfuric acid and ammonia. This shows that *D*_p,th_ is not only determined by the vapor molecule size assumed in the calculations, but follows from the size distribution function, which is affected by both the true molecular size and the evaporation profile.

We also determined *D*_p,th_ from simulations involving different model compounds at different concentrations (Table 4). The simulation results are consistent with the experimental results: *D*_p,th_ is lowest (2.6–2.9 nm) in the simulations involving only SA, and higher with SA and LVOC (3.6–3.7 nm) and with ELVOC and LVOC (3.7–3.8 nm). The only exception is the simulation involving only SA at *C*_SA_ = 8 × 10^6^ cm^−3^, for which *D*_p,th_ is close to the values determined for simulations with ELVOC and LVOC. This indicates that in this simulation, vapor concentration is so low that evaporation is more significant than in other SA simulations. Generally, *D*_p,th_ in simulations and corresponding experiments are very close to each other, with *D*_p,th_ being only slightly (0.1–0.3 nm) higher in the simulations than in the experiments. The agreement is surprisingly good, considering the crude simplifications of the model simulations (*e.g.* the limited number of model compounds and classical evaporation rates). The good agreement may partly be explained by the effect of vapor molecule size on *D*_p,th_, but it also indicates that the approximate size regime in which condensation overcomes evaporation is reasonably represented in the simulations.

**Fig. 10:**
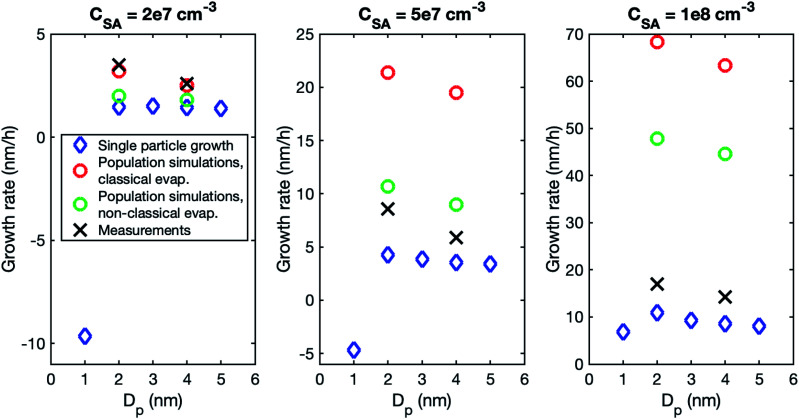
Particle growth rate in a sulfuric acid–ammonia system retrieved for different particle sizes from a single particle growth model (blue diamonds), cluster population simulations with a classical evaporation rate profile (red circles), cluster population simulations with a nonclassical evaporation rate profile (green circles), and from experimental data (black crosses). The different panels show different vapor concentrations. The results from cluster population simulations and experiments correspond to the size ranges of 1–3 and 3–5 or 3–7 nm.

Thus, our results show that stochastic effects are important in the growth of atmospheric particles by sulfuric acid and organic compounds below ∼2.5–3.5 nm. The stochastic effects cannot be considered with single particle growth models, which are based on studying the deterministic condensation mass flux on a particle surface. To demonstrate this, we calculated growth rate from a deterministic condensation flux (see Sect. 2.5), considering the condensation of SA model compound at different concentrations (Fig. 10). In Fig. 10, the single particle model is a valid representation of the growth when the single-particle results (blue diamonds) are close to the population model results with classical evaporation rates (red circles). One should note that in addition to the stochastic effects, the differences between the two approaches stem from other population dynamics effects (such as cluster–cluster collisions) influencing the growth rates derived from population simulations. This applies generally to appearance-time-based growth rates and is relevant to analysis of experimental growth rates in the presence of efficiently clustering chemical compounds.^14^

At *C*_SA_ = 2 × 10^7^ cm^−3^ and *C*_SA_ = 5 × 10^7^ cm^−3^, the growth rate predicted by the single particle growth model is negative for 1 nm particles and positive but lower than growth rate from cluster population simulations and experiments for 2–5 nm particles. This is because at 1 nm, the average cluster evaporation is faster than growth by collisions, and the growth is driven by stochastic effects. At *C*_SA_ = 1 × 10^8^ cm^−3^, growth rates from single particle model vary between 6.9 and 10.9 nm h^−1^ for 1–5 nm particles and are lower than experimental and cluster population simulation results. However, at this high vapor concentration, they are closer to experimental values than growth rates from cluster population simulations, as the latter ones are very high due to the high contribution of cluster–cluster collisions.

Overall, one should be cautious if using single particle growth models to interpret observations on the growth of atmospheric particle population below ∼3 nm. As single particle growth models do not include stochastic effects, they may underestimate the condensational growth at the smallest sizes. This can lead to wrong conclusions on, for example (1) thermodynamic properties of condensing vapors, (2) if the growth can be explained by the observed vapor concentrations or not, (3) if there exist a Kelvin barrier or not.^8^

### 3.5 Discussion

To correctly represent atmospheric particle formation and growth in large-scale models, the knowledge of the growth of sub-10 nm atmospheric particles is essential. Particle growth is commonly studied by assessing particle growth rate from the time-evolution of measured particle size distribution, and growth rates are then used to deduce particle growth mechanisms. In this study, we investigated the factors controlling sub-10 nm particle growth rates in the presence of inorganic and organic vapors, by comparing growth rates determined from chamber experiments to results from molecular-resolution cluster population simulations. The studied experiments involved sulfuric acid, ammonia and/or oxidation products of monoterpenes and our model simulations included either one or two model compounds, representing an inorganic vapor as well as organic vapors with two volatilities (LVOC and ELVOC). Especially, we focused on the size-dependency of particle growth rate, and on the effects of population dynamics and stochastic fluctuations on particle population growth in different atmospheric systems.

In the experiments involving only inorganic vapors (sulfuric acid and ammonia), growth rate was found to decrease with size, similar to previous observations.^36^ Our simulations with a quasi-unary model compound (SA) reproduced this behavior, which is caused by the decreasing vapor molecule size relative to the particle size.^25^ Despite the similar size-dependence of the growth rate, the simulations overestimated the contribution of cluster–cluster collisions to the growth at high vapor concentrations, which results from the evaporation rate profiles assumed in the simulations. Using classical evaporation rates, derived from Kelvin equation, leads to a significant role of cluster–cluster collisions in the growth, which also implies that using these evaporation rates in single-particle condensation models for very small particles is inaccurate. In addition to uncertain evaporation rates, the disagreement between our simulations and the experiments is caused by the limitations of a quasi-unary model compound in representing a binary sulfuric acid–ammonia system. Overall, the sensitivity of particle growth dynamics to evaporation rates highlights the need for accurate estimates of cluster evaporation rates. Especially, there is a lack of quantum chemistry derived evaporation rates for clusters involving large organic molecules, such as HOMs (see Elm *et al.*^67^). In the future, the challenges in identifying and modeling individual organic compounds could be overcome by simulating the behavior of representative functional groups or by using data-driven machine learning approaches.^74^ In addition to computational chemistry methods, inversion modeling approaches can enable retrieving cluster rate constants from experimental data.^75,76^

In the experiments with only organic vapors, growth rate was observed to increase with size, consistently with previous studies.^12^ In the simulations involving two model compounds with different volatilities (LVOC and ELVOC), growth rate increased with size qualitatively similar to the experiments. The increase of growth rate resulted from the increasing contribution of abundant LVOC molecules to growth, which can be qualitatively depicted with nano-Köhler theory, describing the activation of particles to growth by oxidized organic vapors.^32^

In the experiments involving both sulfuric acid and organic vapors, growth rate either decreased with size or stayed close to constant. We were unable to reproduce this behavior with our simulations and thus it requires further investigation. In all the simulations involving SA and LVOC, growth rate increased with size, but the strength of the increase depended on LVOC concentration. These results illustrate that nano-Köhler type activation, with clearly accelerating growth rate, occurs only under specific vapor concentrations and saturation ratios. Previously, the acceleration of particle growth rate has been interpreted to show the size at which organic vapors start to contribute to growth.^31^ Our results indicate that while the increase of growth rate with size can be connected to the increasing contribution of organic vapors to the growth, oxidized organic vapors can also contribute to the growth in a situation where the growth rate does not increase with size.

To study the importance of stochastic collisions and evaporations in particle growth, we determined the threshold size *D*_p,th_ below which stochastic effects are nonnegligible^8^ in different experiments and simulations. We found that stochastic effects can be important in the growth of atmospheric particles by sulfuric acid and organic compounds below ∼3 nm, and the exact threshold size depends on the concentrations and properties (*i.e.* evaporation rates and molecule size) of the vapors. When the observed growth rates are interpreted using a condensation model, based on studying a vapor mass flux on a single particle, the limitations of this modeling approach should be recognized. Especially at particle sizes below 3 nm, stochastic collisions may enhance particle growth significantly. Thus, neglecting them can lead to wrong conclusions on particle growth mechanisms.

Generally, our results on the importance of the effects of particle-population dynamics on observed growth rates imply that particle growth rate should be viewed to describe the growth of the whole particle population, instead of a diameter growth rate of a single representative particle. When modeling particle growth, the stochastic fluctuations and population dynamics effects can be considered by using particle population simulations.^9,10,73^ Regarding measured growth rates, GDE-based methods^29,77,78^ to determine growth rate should be preferred over simpler ones, such as the appearance time method. This is because GDE-based methods can separate the effects of condensation and coagulation on particle growth rate, although they neglect stochastic effects. Moreover, they can retrieve both size- and time-dependence of growth rates, which makes it easier to evaluate the effect of vapor time-dependence on the behavior of the growth rate. Recently, Ozon *et al.*^78^ introduced a method based on applying a Kalman smoother to a finite difference solution of GDE that can also provide the uncertainty range for measured growth rate. The uncertainties of sub-10 nm particle size distribution measurements are significant,^34^ which propagates in the growth rate values and should thus be considered when interpreting particle growth observations.

In the broader view, our results suggest that instead of only investigating particle growth rates to understand the dynamics of a growing particle population, the focus should be shifted towards directly studying the evolution of particle size distribution and developing methods for this. For this, models accurately simulating the time-evolution of particle population and reliable measurements of the particle size distribution below 10 nm are also needed.

## 4. Conclusions

**Fig. 11:**
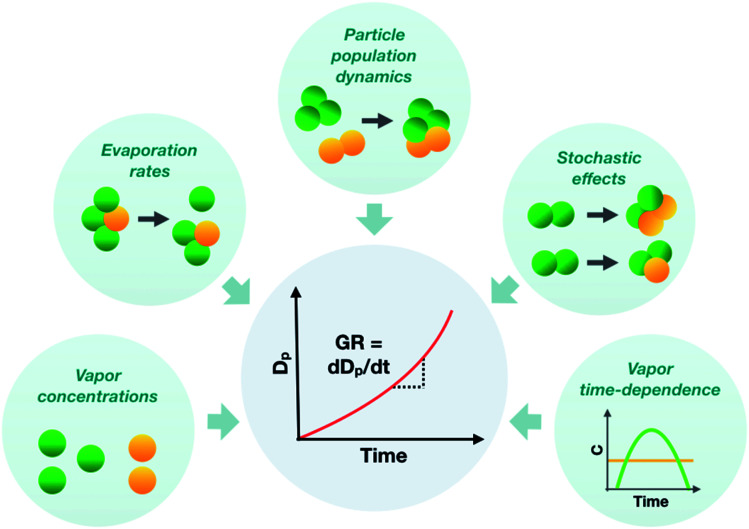
Schematic figure illustrating different factors influencing the observed sub-10 nm particle growth rate (GR).

To represent atmospheric particle growth accurately in large-scale models, the observations on particle growth should be correctly interpreted. In this study, we unravel the factors controlling the observed growth rates of sub-10 nm atmospheric particles, using cluster population simulations and chamber experiments. Fig. 11 summarizes the different factors affecting the observed particle growth rates according to our results. They include the concentrations of condensable vapors, their evaporation rate profiles, their time-dependent variation as well as stochastic fluctuations and particle population dynamics effects, such as cluster–cluster collisions. Many approaches commonly used to interpret growth rate observations are unable to consider all these effects, which can lead to a biased view on the properties of condensing vapors and particle growth mechanisms. Therefore, to draw conclusions on observed particle growth rates, especially in the sub-10 nm size range, new data-analysis approaches and particle population modeling are needed.

## Author contributions

Conceptualization: JK, DS, TO, KL, IR; methodology: JK, TO, LA; formal analysis: JK, DS, CY; investigation: JK, DS, CY, LD, LA, MS, KL; writing – original draft preparation: JK, DS, TO, KL, IR; writing – review and editing: all authors; visualization: JK.

## Data availability

The particle size distributions measured with the DMA train are available at: https://doi.org/10.5281/zenodo.6362724 (Stolzenburg, 2022).^79^ The simulation data are available at: https://doi.org/10.5281/zenodo.6370141 (Kontkanen *et al.*, 2022).^80^

## Conflicts of interest

There are no conflicts of interest to declare.

## Supplementary Material

EA-002-D1EA00103E-s001
