# Supplementary material for: What controls the observed size-dependency of the growth rates of sub-10 nm atmospheric particles?
Source: Environ Sci Atmos. 2022 Mar 23;2(3):449–68. doi: 10.1039/d1ea00103e (PMC9119032; doi:10.1039/d1ea00103e)
Supplement: EA-002-D1EA00103E-s001 [file EA-002-D1EA00103E-s001.pdf]

Electronic Supplementary Material (ESI) for Environmental Science: Atmospheres.
This journal is © The Royal Society of Chemistry 2022

Supplementary information

Table S1. Precursor vapor concentrations in the studied experiments. HOM concentrations are shown separately for HOM monomers and dimers not containing nitrogen ($HOM_{non\text{-}nit,mon}$ and $HOM_{non\text{-}nit,dim}$) and for those containing nitrogen ($HOM_{nit,mon}$ and $HOM_{nit,dim}$).

Exp. no.	CLOUD run no.	Trace gases added	H_2SO_4 (cm^{-3})	$HOM_{non\text{-}nit,mon}$ (cm^{-3})	$HOM_{nit,mon}$ (cm^{-3})	$HOM_{non\text{-}nit,dim}$ (cm^{-3})	$HOM_{nit,dim}$ (cm^{-3})
1	1764.11	AP, DC, SO_2, NO_x, NH_3	7.8×10^6	1.3×10^7	1.2×10^7	1.5×10^6	1.7×10^6
2	1764.22	AP, DC, SO_2, NO_x, NH_3	7.9×10^6	1.1×10^7	1.1×10^7	1.4×10^6	1.5×10^6
3	1764.26	AP, DC, SO_2, NO_x, NH_3	9.7×10^6	1.1×10^7	1.1×10^7	1.3×10^6	1.5×10^6
4	1767.08	AP, DC, SO_2, NO_x, NH_3	8.3×10^6	7.7×10^6	7.5×10^6	1.4×10^6	1.6×10^6
5	1768.08	AP, NO_x	-	1.7×10^7	8.9×10^6	4.1×10^6	3.7×10^6
6	1769.02	AP, NO_x	-	1.5×10^7	4.2×10^6	3.8×10^6	1.2×10^6
7	1769.06	AP, NO_x	-	1.5×10^7	4.1×10^6	3.9×10^6	1.2×10^6
8	1769.08	AP, NO_x	-	1.4×10^7	3.8×10^6	3.7×10^6	1.1×10^6
9	1952.01	SO_2, NH_3	2.0×10^7	-	-	-	-
10	1952.04	SO_2, NH_3	4.7×10^7	-	-	-	-
11	1952.06	SO_2, NH_3	1.1×10^8	-	-	-	-

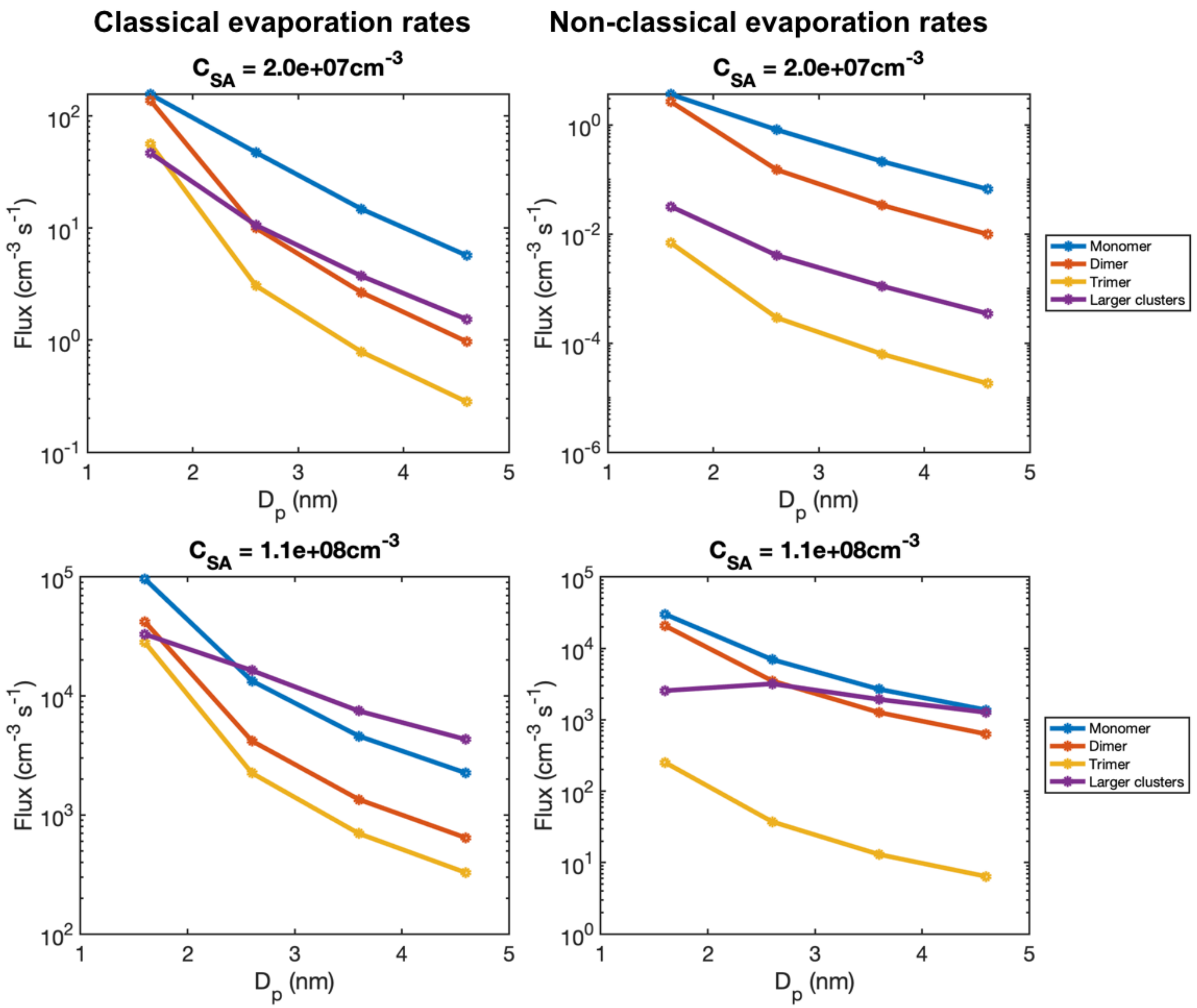

Fig S1. The net particle flux due to vapor monomers and clusters past different threshold sizes in simulations with SA at two different concentrations (C_{SA} = 2.0 × 10^7 and 1.1 × 10^8 cm^{-3}). The values are at the steady state.

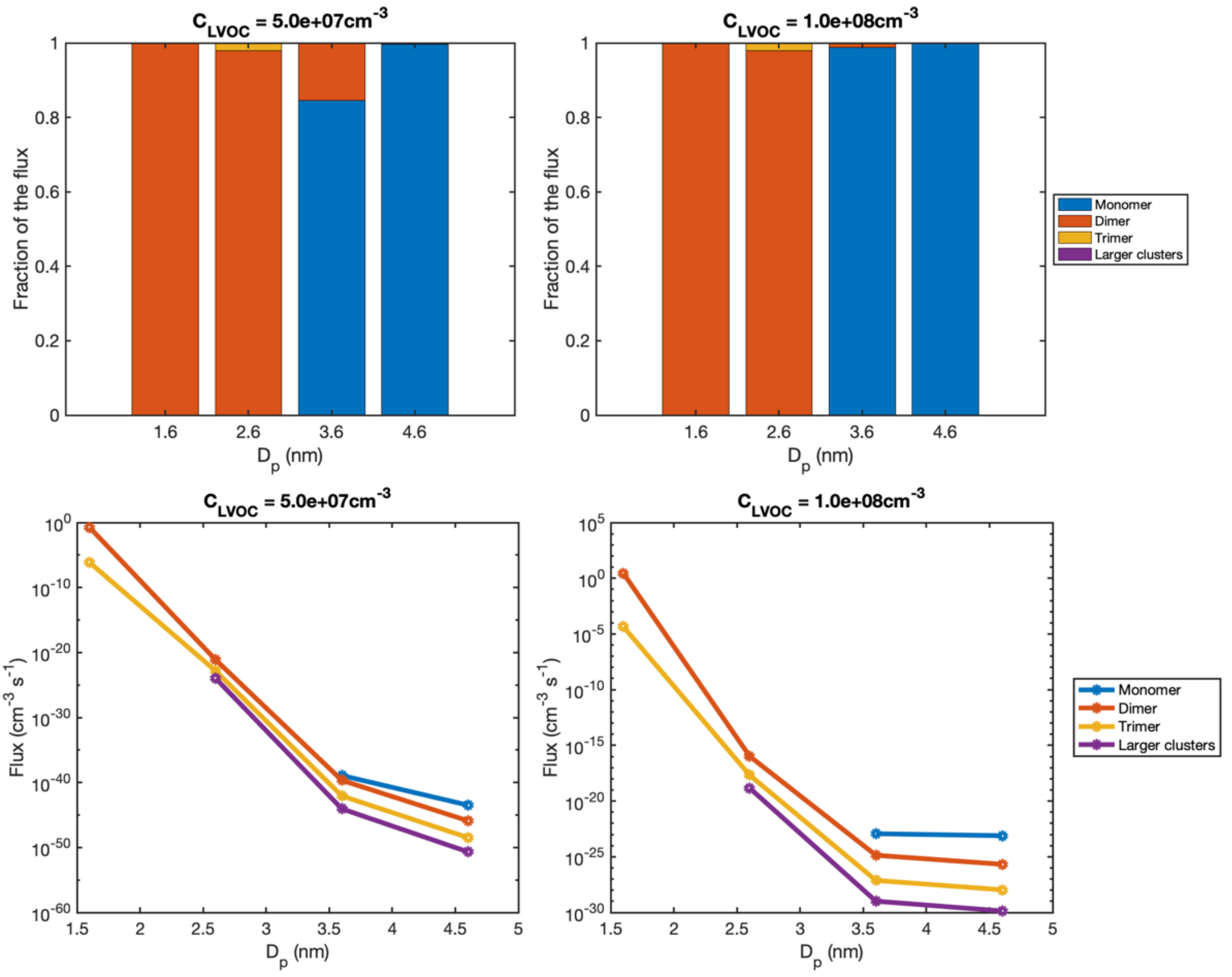

Figure S2. Contribution of vapor monomers and clusters to the net particle flux past different threshold sizes in simulations with LVOC at two different concentrations ($C_{LVOC} = 5 \times 10^7$ and 1×10^8 cm^{-3}). The top panels show the fractions of the flux, and the bottom panels show the absolute fluxes. For monomer, the absolute flux is negative at the smallest sizes and therefore not shown. The values are at the steady state.

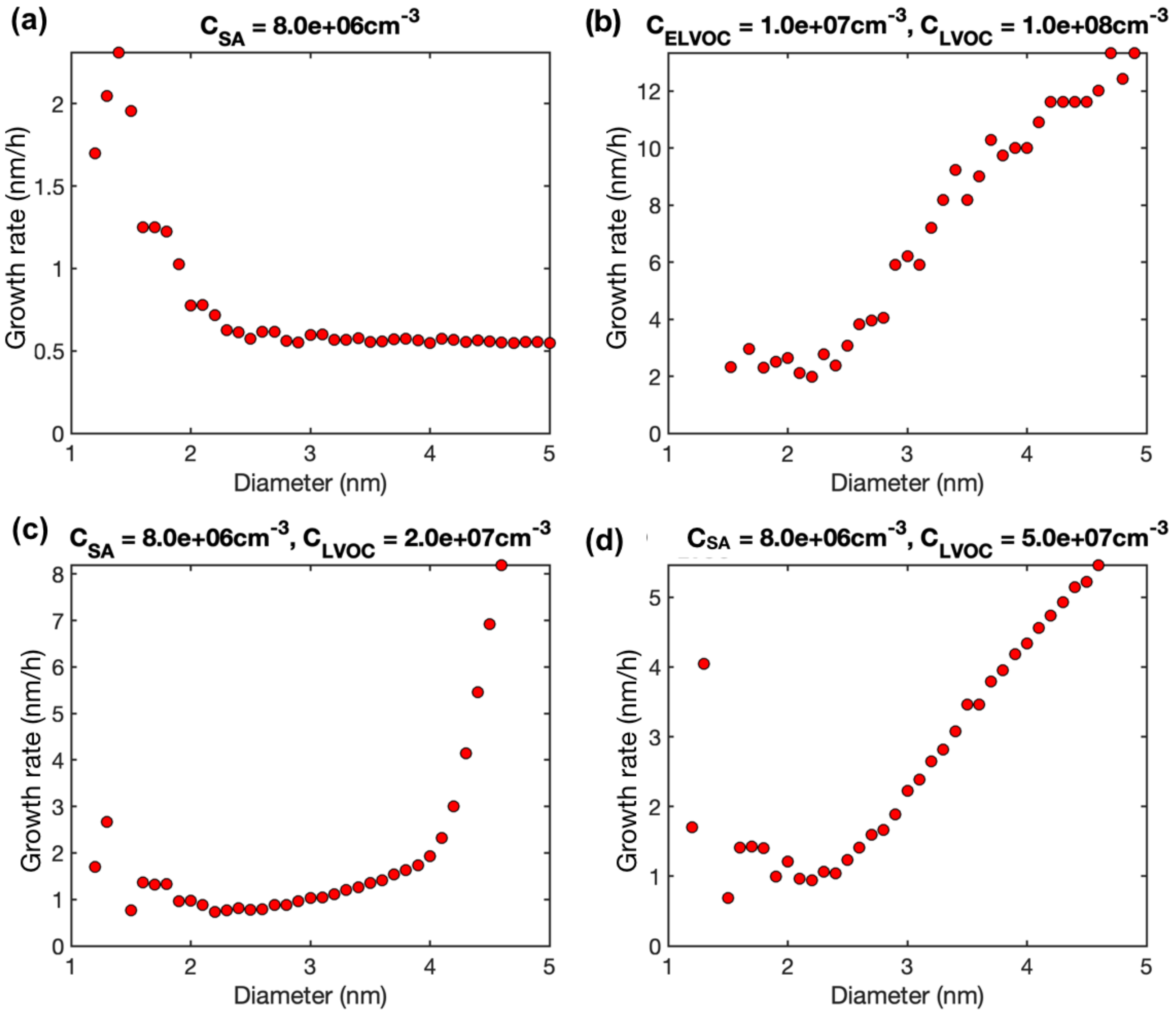

Figure S3. Growth rate as a function of particle size, obtained as a slope of (D_p, t_{app}) data (see Fig. 7), in simulations with different model compounds : (a) SA at $C_{SA} = 8.0 \times 10^6$ cm^{-3}, (b) LVOC and ELVOC at $C_{LVOC} = 1 \times 10^8$ and $C_{ELVOC} = 1 \times 10^7$ cm^{-3} (c) SA and LVOC at $C_{SA} = 8 \times 10^6$ and $C_{LVOC} = 2 \times 10^7$ cm^{-3}, (d) SA and LVOC at $C_{SA} = 8 \times 10^6$ and $C_{LVOC} = 5 \times 10^7$ cm^{-3}. Note that in (c), the sharp increase of growth rate above 4 nm is connected to the cluster concentrations not reaching the steady state in the end of this simulation.

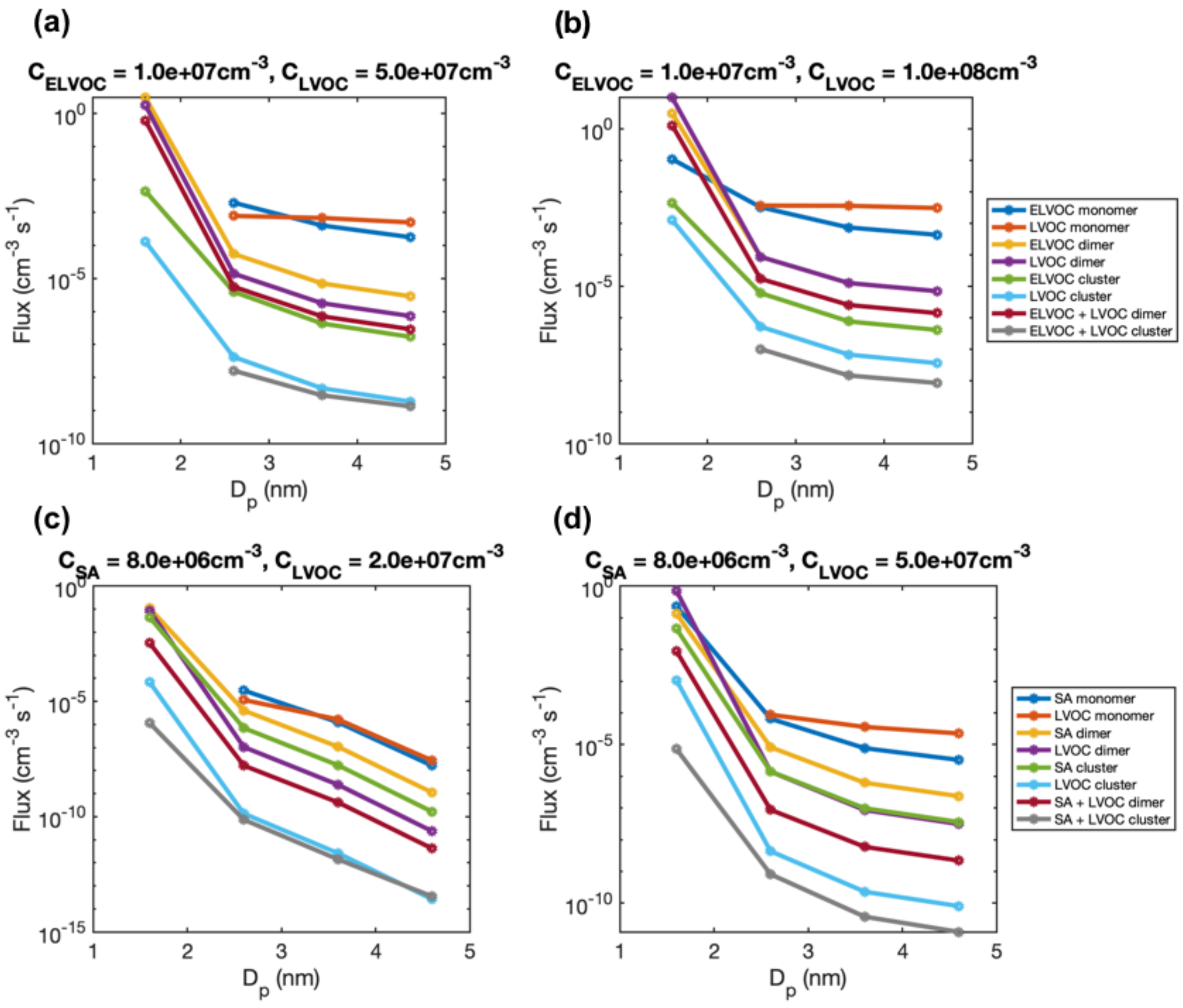

Figure S4. Particle fluxes due to vapor monomers and clusters past different threshold sizes in simulations with (a) LVOC and ELVOC at $C_{ELVOC} = 10^7$ cm^{-3} and $C_{LVOC} = 5 \times 10^7$ cm^{-3}, (b) LVOC and ELVOC at $C_{ELVOC} = 10^7$ cm^{-3} and $C_{LVOC} = 10^8$ cm^{-3}, (c) SA and LVOC at $C_{SA} = 8 \times 10^6$ and $C_{LVOC} = 2 \times 10^7$ cm^{-3}, (d) SA and LVOC at $C_{SA} = 8 \times 10^6$ and $C_{LVOC} = 5 \times 10^7$ cm^{-3}. The values are at the steady state.
